# Screening and Risk Assessment of Hepatitis B and C Among Medical Students and Non-teaching Staff of Services Institute of Medical Sciences, Lahore

**DOI:** 10.7759/cureus.94170

**Published:** 2025-10-09

**Authors:** Rabiah Mahwish, Fatima Abbas, Muhammad Qasim Ali, Muhammad Mohamin, Shaheer Afzal

**Affiliations:** 1 Community Medicine, Khawaja Muhammad Safdar Medical College, Sialkot, PAK; 2 Medicine, Services Institute of Medical Sciences, Lahore, PAK

**Keywords:** hepatitis b, hepatitis c, medical students, risk assessment, screening

## Abstract

Objective

This study aims to screen and assess the risk of hepatitis B and C among medical students and non-teaching staff at Services Institute of Medical Sciences (SIMS), Lahore.

Material and methods

This cross-sectional descriptive study was conducted at a screening camp using non-probability convenience sampling from September 7 to October 31, 2023. A total of 385 participants provided informed consent for both hepatitis screening and completing a modified form of the Hepatitis Risk Assessment Tool from the Centers for Disease Control and Prevention (CDC). Participants included medical students and non-teaching staff, regardless of comorbidities, who were not previously diagnosed with hepatitis B or C. Screening for hepatitis B and C was conducted using self-financed kits.

Results

Among the 385 participants, none tested positive for hepatitis B, while four participants tested positive for hepatitis C. The mean age of participants was 23.98 ± 6.686 years. Risk factor analysis revealed that 11 (3%) participants had a history of blood transfusion, and three (0.8%) reported prior dialysis. A total of 123 (32%) participants had undergone liver function tests (LFTs), with 34 (8.8%) showing abnormal results. Additionally, 216 participants had not received hepatitis B vaccination, and only 98 had completed the vaccination schedule. Furthermore, 51 (13%) participants had a history of injecting drugs.

Conclusion

Approximately half of the participants were vaccinated against hepatitis B, and a minimal proportion had elevated risks for hepatitis B and C. None tested positive for hepatitis B, and a small number were positive for hepatitis C, suggesting a differing prevalence of the two infections. Hepatitis screening should be routinely conducted in educational institutions, particularly for individuals with significant risk factors.

## Introduction

Hepatitis B and C are viral infections that target the liver. These viruses spread through shared needles, syringes, other injection equipment, perinatal transmission, and sexual contact. In men, exposure to unsterilized barber equipment and improperly screened blood is a significant cause of transmission, while gynecological surgical procedures and blood transfusions are common causes in women [1,2]. As of 2022, according to the World Health Organization, about 254 million people are living with hepatitis B, and 50 million people have been diagnosed with hepatitis C, with 6,000 people being diagnosed with viral hepatitis every day [3]. In Pakistan, the overall prevalence of hepatitis B is 2.5%, while hepatitis C stands at 4.8%, resulting in a combined infection rate of 7.6% in the general population [4].

Although anyone can contract hepatitis B or C, certain groups are at greater risk, including infants born to infected mothers, individuals who inject drugs or share needles, sex partners of people with hepatitis B, individuals with sexually transmitted infections or HIV, and healthcare workers exposed to blood. The Centers for Disease Control and Prevention (CDC) recommends periodic screening for these high-risk populations [5]. Screening is also advised for all adults aged 18 and older at least once in their lifetime, as well as during each pregnancy for pregnant women [6]. Symptoms of hepatitis B and C may not appear for years or even decades after infection. Even without symptoms, these viruses can still damage the liver. Early detection through screening helps reduce complications such as cirrhosis, liver cancer, and deaths resulting from chronic infection [6,7].

Research indicates a 5% prevalence of chronic hepatitis B among healthcare workers [8]. Another study showed an 11% prevalence of hepatitis B among medical students, with a higher infection rate in clinical students [9]. In a study conducted among students in a university in Nigeria, six out of 1,572 students tested positive for hepatitis C, resulting in an overall prevalence of 0.40% [10].

The aim of this study is to determine the prevalence of hepatitis B and C among medical students and non-teaching staff at Services Institute of Medical Sciences (SIMS), Lahore, through rapid diagnostic screening and assess the risk factors associated with hepatitis B and C using a structured risk assessment tool and, in the process, evaluate the hepatitis B vaccination status of the participants. The findings will contribute to preventive measures, early treatment, and future studies on disease prevalence.

## Materials and methods

A descriptive cross-sectional study was conducted at a hepatitis screening camp held at the Services Institute of Medical Sciences (SIMS), Lahore, from September 7 to October 31, 2023. The camp aimed to raise awareness among medical students regarding hepatitis screening, as they are particularly susceptible to acquiring hepatitis due to occupational exposure. Ethical approval was obtained from the Institutional Review Board (IRB) of Services Hospital, Lahore/SIMS (approval number: IRB/2023/1206/SIMS) to conduct the survey and screen participants.

The sample size was calculated using the standard formula for estimating proportions in a cross-sectional study, assuming a 95% confidence level, 5% margin of error, and an expected prevalence of 50%. The minimum required sample size was 385 participants.

Using a non-probability convenience sampling technique, 385 participants, including medical students and non-teaching staff of all age groups, were enrolled after providing informed consent. Participants were recruited during the hepatitis screening camp through on-site announcements, posters, and direct outreach by medical volunteers. Individuals previously diagnosed with hepatitis B or C were excluded. While convenience sampling allowed for rapid recruitment, it carries limitations such as potential bias, while the second recorded medical history. A modified version of the CDC’s Hepatitis Risk Assessment Tool, consisting of nine items, was used to evaluate risk levels among participants. However, since it was not formally validated as a diagnostic tool, it may introduce selection bias and reduce the generalizability of the findings.

Data collection involved an interviewer-administered questionnaire comprising two parts. The first captured demographic data; it is widely utilized for public health screening and risk stratification purposes. The second part recorded medical history and assessed hepatitis risk using a modified version of the CDC's Hepatitis Risk Assessment Tool, consisting of nine items [11]. Those scoring above the mean were categorized as “at risk,” while those scoring below were considered “not at risk.” The mean score was calculated using SPSS version 22 (IBM Corp., Armonk, NY).

Screening for hepatitis B and C was performed using commercially available rapid diagnostic kits, such as Determine™ HBsAg 2 (Abbott Laboratories, Abbott Park, IL) and hepatitis C virus (HCV) antibody tests. These kits offer quick turnaround times, as results were delivered within 10 minutes of sample collection, and are suitable for use in low-resource settings. The sensitivity of these kits for hepatitis B surface antigen (HBsAg) detection ranges from 90% to 100%, with specificity between 98% and 100%. For hepatitis C antibody (HCV-Ab) detection, sensitivity typically ranges from 92% to 99%, and specificity from 97% to 100%, making them reliable tools for point-of-care screening.

All screening results were kept strictly confidential. Participants were also educated about hepatitis B and C, including transmission routes, risk factors, and preventive measures, to enhance awareness and promote safer health practices.

Data were entered and analyzed using SPSS version 26. Descriptive statistics (mean and standard deviation) were applied to quantitative variables, while frequencies and percentages summarized qualitative data.

## Results

**Figure 1:**
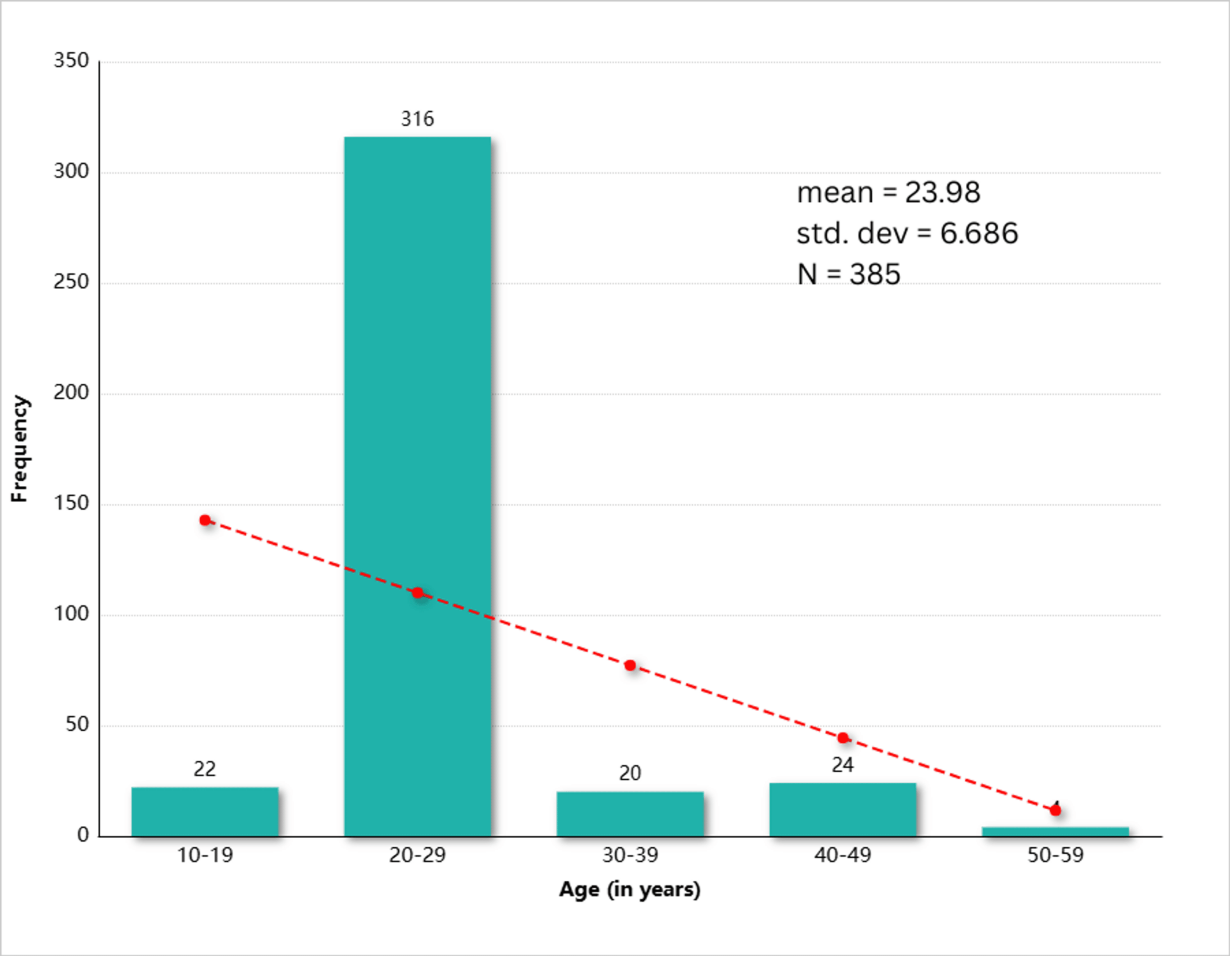
Age of the respondents in years

A total of 385 respondents participated in this study and were screened for hepatitis B and C. The mean age of the participants was 23.98 + 6.686, as shown in Figure 1. There were 207 (53.8%) male respondents and 178 (46.2%) female respondents. Among the study participants, 47 (12.2%) were married and 338 (87.8%) were unmarried.

As shown in Table 1, of the 385 participants screened, none of them screened positive for hepatitis B, whereas only four participants were positive for hepatitis C. Various risk factors of the participants for acquiring the disease were assessed, and it was found that 11 (3%) patients had received blood transfusions, and three (0.8%) of them had a history of dialysis. A total of 123 (32%) participants had done a liver function test (LFT) as indicated by their doctor for various medical reasons, and 34 (8.8%) had deranged results.

**Table 1:** Characteristics of the respondents LFT: liver function test

Characteristics	Frequency (number (%))	
	Yes	No
Diagnosed with hepatitis B on screening	0 (0)	385 (100)
Diagnosed with hepatitis C on screening	4 (1)	381 (99)
Ever received a blood transfusion	11 (3)	374 (97)
Ever donated blood	97 (25)	288 (75)
Received any type of injection (medical or non-medical)	115 (30)	270 (70)
History of any major surgery	52 (13)	333 (87)
History of any dental procedure	47 (12)	338 (88)
History of any organ transplant	24 (6)	361 (94)
History of dialysis	3 (0.8)	382 (99)
Ever done an LFT	123 (32)	262 (68)
If done, was it deranged	34 (8.8)	351 (91.2)

About 211 of the participants had not had adult vaccination against hepatitis B, whereas only 98 of the remaining participants had completed the vaccination schedule against hepatitis B, as detailed in Table 2.

**Table 2:** Vaccination status of the participants against hepatitis B

Vaccination status	Frequency (number (%))
Not vaccinated	211 (54.8)
Completed vaccination (third dose)	98 (25.5)
Second dose administered	31 (8.1)
First dose administered	45 (11.7)
Total	385 (100)

None of the respondents were diagnosed with HIV or had a history of having sex with the same gender, but two of them (1%) had been diagnosed with a sexually transmitted disease (STD). About five (1%) participants are currently living with someone diagnosed with hepatitis, whereas 13 (3%) previously lived with persons diagnosed with the disease mentioned. About 51 (13%) participants had a history of unintentional exposure to injection equipment (Table 3).

**Table 3:** Risk assessment tool for hepatitis B and C STD: sexually transmitted disease, HIV: human immunodeficiency virus, AIDS: acquired immunodeficiency syndrome

Risk assessment	Frequency (number (%))	
	Yes	No
Diagnosed with a clotting disorder	7 (2)	378 (98)
Diagnosed with chronic liver disease	0 (0)	385 (100)
Currently live with someone who is diagnosed with hepatitis B	5 (1)	380 (99)
Previously lived with someone who has been diagnosed with hepatitis B	13 (3)	372 (97)
Diagnosed with an STD	2 (1)	383 (99)
Diagnosed with diabetes	8 (2)	377 (98)
Diagnosed with HIV/AIDS	0 (0)	385 (100)
Have sexual encounters with the same sex	0 (0)	385 (100)
Unintentional exposure to used injection equipment	51 (13)	334 (87)

## Discussion

The prevalence of hepatitis B and C is rising in developing countries compared to developed nations. The mean age of the participants in this study was 23.98 ± 6.686 years, with 207 (53.8%) male participants and 178 (46.2%) female participants. These demographics align with a study conducted across colleges in Saudi Arabia, where 16,570 students participated (9,852 male participants (59.4%) and 6,718 female participants (40.6%)), with a mean age of 21 years divided into two age groups, 18-21 and 22-30 years [12].

Hepatitis B and C remain common infectious diseases in third-world countries, with hepatitis C being more prevalent than hepatitis B. This trend was observed in a study conducted in a town near Islamabad and can be attributed to immunization programs for newborns and adolescents, which have reduced the overall incidence of hepatitis B [13,14]. In this study, four (1%) participants out of 385 screened positive for hepatitis C, while none tested positive for hepatitis B. Among the participants, only 98 were fully vaccinated against hepatitis B, and 211 had never received any form of vaccination. These findings are similar to a study conducted in a medical college in Kathmandu, Nepal, from July 6 to July 14, 2020, where 37% of students were vaccinated [15]. All participants who tested positive for hepatitis C were non-students with a history of sexually transmitted infections, aligning with CDC research indicating higher prevalence in such groups [16]. Additionally, all positive cases reported a history of repeated drug injections, a known risk factor supported by other studies [17].

Blood transfusion is another critical risk factor for hepatitis C. In this study, 11 participants reported a history of blood transfusion. Previous research estimates the prevalence of hepatitis-positive blood transfusions at around 4.7% [18]. Of the participants who tested positive for hepatitis C, three had a history of dialysis. This finding aligns with another study, where 3% of 1,200 dialysis patients were positive for hepatitis C [19].

Deranged liver function tests (LFTs) were observed in 34 (8.8%) participants, while seven (2%) participants had significantly abnormal results. A retrospective study conducted in Victoria on patients with clotting disorders found that 76% of patients had antibodies to hepatitis C, and 65% showed markers of hepatitis B infection. That study also demonstrated a strong correlation between hepatitis C antibodies and raised transaminase levels, further highlighting the impact of viral hepatitis on liver health [20].

This study was limited to a single educational institution, with participants primarily comprising young, educated individuals (medical students and non-teaching staff) who may have had greater awareness of hepatitis-related risks, reducing generalizability to the broader population. The use of non-probability convenience sampling introduces selection bias, and reliance on self-reported data may be subject to recall bias. Diagnostic accuracy metrics such as predictive values were not reported, and detailed statistical methods were not fully described. Potential confounders such as socioeconomic status and prior healthcare access were not controlled for, and the modified CDC Hepatitis Risk Assessment Tool used for screening has not been formally validated in the local context. Additionally, the cross-sectional design limits causal inference and follow-up, which restricts the ability to assess long-term outcomes or behavioral changes. To address the growing prevalence of hepatitis B and C in developing countries like Pakistan, more extensive screening programs targeting both medical students and the general population are essential.

## Conclusions

Of the participants, 44% were vaccinated against hepatitis B, and only a small proportion were identified as having increased risk factors for hepatitis B and C. None of the participants tested positive for hepatitis B, and only a few tested positive for hepatitis C, highlighting the differing prevalence rates of the two infections. Routine screening for hepatitis should be implemented in schools and colleges, with a focus on individuals with significant risk factors to enable early detection and intervention.
